# The Mediation Role of Fatness in Associations between Cardiorespiratory Fitness and Blood Pressure after High-Intensity Interval Training in Adolescents

**DOI:** 10.3390/ijerph19031698

**Published:** 2022-02-01

**Authors:** Jarosław Domaradzki, Dawid Koźlenia, Marek Popowczak

**Affiliations:** 1 Department of Biostructure, Faculty of Physical Education and Sport, Wroclaw University of Health and Sport Sciences, al. I.J. Paderewskiego 35, 51-612 Wroclaw, Poland; jaroslaw.domaradzki@awf.wroc.pl; 2 Department of Team Sports Games, Faculty of Physical Education, Wroclaw University of Health and Sport Sciences, al. I.J. Paderewskiego 35, 51-612 Wrocław, Poland; marek.popowczak@awf.wroc.pl

**Keywords:** adolescents, high-intensity interval training, Tabata protocol, body fat, blood pressure, cardiorespiratory fitness, physical education lessons, mediation analysis

## Abstract

Background: Low cardiorespiratory fitness (CRF), elevated blood pressure (BP), and high fatness are cardiovascular disease risk factors. It remains unknown how fatness affects the influence of CRF on BP. Therefore, the aim was to examine whether the associations between baseline CRF and HIIT-postintervention resting BP were mediated through fatness in adolescents. Material and methods: The sample (n = 64) comprised 28 boys 36 girls aged 16 years. The analysed factors were fitness index- reflecting CRF, body fat mass (BFM), fat mass index (FMI), and body mass index (BMI). Bootstrapped mediation procedures were performed. The mediation analysis was conducted concerning the sex moderation effect. Results: The mediation effect of sex was confirmed; therefore, mediations models were designed separately for boys and girls. The results showed a statistically significant inverse relationship between baseline CRF and post-intervention systolic blood pressure (SBP) in both sexes (boys: B = −0.603, *p* = 0.010; girls: B = −0.394, *p* = 0.037). The relations between CRF and SBP revealed the mediation effect of the BFM and the FMI only in boys. Conclusions: Fatness mediated associations between CRF and SBP in boys. Therefore, both CRF and fatness are necessary to maintain positive results of the BP revealed in normal SBP ranges after HIIT.

## 1. Introduction

The worldwide prevalence of obesity, hypertension, and low cardiorespiratory fitness (CRF) are well-documented [1,2,3]. Very close connections between fatness, elevated blood pressure, and low, inadequate cardiorespiratory fitness, reported many times [4,5], strengthen the adverse influence of all mentioned parameters on health, leading to cardiovascular diseases (CVD). The global public health problem is related to CVD appearing increasingly in childhood and adolescents [6,7].

A high level of fatness is a risk factor for elevated blood pressure (BP) in adolescents [8]. Contrary to fatness, an inverse relationship between CRF and BP has been proven [9,10]. The triad of associations between the variables mentioned above is filled by the relationship between higher CRF with decreased body fat mass (BFM) [11]. Therefore, deepening the knowledge about risk factors of elevated blood pressure and how to lower BP is urgent and the necessity to tackle its dangerous consequences, particularly in adolescents.

Physical activity (PA) is an effective way to decrease whole body fat and resting BP and improve CRF [12,13,14]. The reluctance of adolescents to engage in PA is a very significant problem and the main cause of the current global pandemic of physical inactivity [15]. The problem of how to oblige young people to engage in physical exercise is one of the main focuses of scientific research. The solution seems to be physical education (PE) lessons and the implementation of programs that increase the intensity of exercises [16].

One of the most effective methods for improving fitness and body composition is high-intensity interval training (HIIT). Evidence from previous studies support a positive influence on body mass index (BMI), waist-to-hip ratio (WHR), and whole BFM [17,18,19]. The reported effects were a reduction in fat and resting BP and an increase in CRF after HIIT intervention [20,21,22].

To date, little is still known about the possible ways HIIT affects BP, especially the role of CRF and BF at the baseline (preintervention) concerning effects in resting BP after the intervention, though single studies have suggested that whole bodyweight could mediate the relationship between CRF and BP [23,24,25], or even BF could mediate BP in a distant perspective [26]. According to the authors’ best knowledge, there are no studies reporting body fat mass as a potential mediator in associations between CRF and postintervention BP.

Thus, considering the relationship between cardiorespiratory fitness, fatness, and resting blood pressure after the intervention of the high-intensity interval training [8,9,10,11], to elucidate whether fatness plays a role as a potential mediator in associations between CRF and BP would aid to explain underlying mechanisms and confirm, in another way, that HIIT intervention is a valuable preventive method, affecting cardiovascular health in adolescents. 

Therefore, the main aim of the work was to examine whether the associations between CRF and postintervention resting BP (both SBP and DBP) were mediated through body fatness in adolescents. Specifically, we put forward four detailed questions: (1) Did the resting BP after HIIT intervention depend on CRF at baseline? (2) Did the CRF associated with fatness affect resting BP after HIIT intervention? (3) Did the effect of CRF on resting BP after intervention disappear after including fatness (BMI, FMI, BFM) into the model—other words: could fatness indices act as a mediator in the relationship between CRF and BP? (4) Did sex moderate such potential associations? Although the main mediator tested in the mediation model was body fat mass (BFM in kilograms), the effectiveness of the fat mass index (FMI) compared to body mass index (BMI) as the mediators were also tested. 

**Figure 1:**
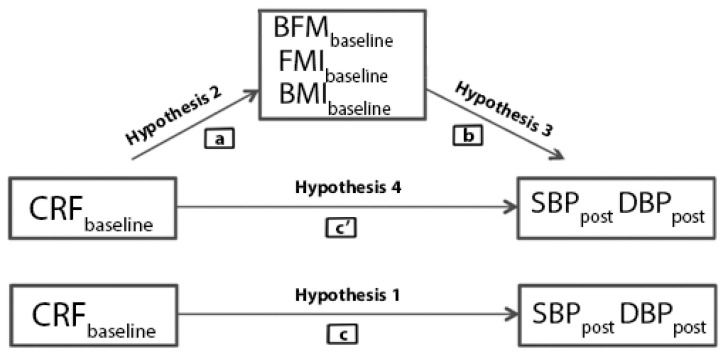
Hypothesized mediation model: an indirect effect of preintervention cardiorespiratory fitness (CRFbaseline) (paths: ab) on blood pressure after intervention (SBPpost, DBPpost), through preintervention fatness (BFMbaseline, FMIbaseline, BMIbaseline) mediation (paths a and b), a direct effect of baseline CRF on SBP and DBP (path c’) and the total effect (path c) of the independent variable on the dependent variable.

Based on the procedure proposed by Baron and Kenny [27], the following hypotheses should be tested: Hypothesis 1—Baseline CRF improves resting postintervention blood pressure (SBP and DBP), decreasing both measurements; Hypothesis 2—This total effect would be mediated by body fat mass (or fat mass index and body mass index), meaning CRF affects (modify) fatness; and—Hypothesis 3—In consequence, fatness (BFM, FMI, BMI) improves BP, with -Hypothesis 4—The direct effect of CRF on BP disappearing in the presence of mediating variable. A graphical presentation of the presented hypothetical model is shown in Figure 1. 

## 2. Materials and Methods

This study was part of the project “Physical activity and nutritional education in preventing civilization diseases—theoretical aspects and practical implications for the secondary school physical education program”. The project was a school-based prospective study carried out in one of the secondary schools in Wroclaw—a city in the southwest of Poland. The project aimed to search the methods supporting standard education in increasing the intensity of exercises to adjust body composition, motor performance, cardiorespiratory fitness, and cardiovascular functions. High-intensity interval training was an intervention program implemented into PE lessons. On the other hand, part of the project was an educational campaign on the proper nutrition of adolescents. A detailed description of the sampling, full number of participants, recruitment approaches, intervention design, and data collection, in addition to several analyses of the effectiveness of HIIT on CRF, body composition, musculoskeletal fitness, including comparisons between experimental and control groups, effects of HIIT in groups with different fat mass level and between normal and hypertensive adolescents, were published elsewhere [20,28]. 

### 2.1. Participants in This Study

The study sample included 73 individuals who took part in the HIIT intervention program. The participants were in three classes, randomly assigned to the experimental group (out of six classes, where three were control groups). Regarding aim, the results only experimental group were used in this work. Furthermore, we decided to study the mediation effect of fatness in the relationship between baseline preintervention CRF and postintervention SBP and DBP only in adolescents with no hypertension. Therefore, the results of nine individuals were excluded before starting mediation analysis. In detail, three boys and six girls presented values of SBP or DBP above the threshold of hypertension (see identifying individuals with hypertension).

The final analysis involves 64 adolescent students—28 boys aged 16.17 ± 0.30 and 36 girls aged 16.11 ± 0.39. The participants were volunteers, and they could quit at any time. The students and their parents or guardians were informed about the objectives and rules of the study. The school principal, parents and study participants gave written informed consent before participation. 

### 2.2. Procedures

Measurements were taken before (baseline variables) and after the 10-week intervention (postintervention) in one day from 8:00 a.m. to 1:00 p.m. Participants were asked to excrete, avoid physical activity, avoid excessive drinking of liquids, and keep their typical morning patterns directly before measurements.

### 2.3. Anthropometric and Body Fat Measurements

Body height was measured with an accuracy of 0.1 cm, using anthropometers (GPM Anthropological Instruments, DKSH Ltd., Zurich, Switzerland). Bodyweight and body fat mass (BFM) was measured using an InBody230 body composition analyzer (InBody Co., Ltd., Cerritos, CA, USA), which was positively validated as a reliable tool. High intraclass correlation coefficients indicated very high reliability for BF% (≥0.98), FM (≥0.98), and FFM (≥0.99), as well as the low standard error of measurement [29]. 

Based on the values of height and weight obtained, the index of relative body mass—BMI (kg/m^2^)—was calculated using the following formula:

(1) $$\mathrm{BMI}={\frac{\mathrm{body}\;\mathrm{mass}\;\left[\mathrm{kg}\right]}{{\mathrm{body}\;\mathrm{height}\;[m}^{2}]}}^{}$$

Based on the body fat mass results—fat mass index (FMI) was calculated from a similar formula such as BMI:

(2) $$\mathrm{FMI}={\frac{\mathrm{body}\;\mathrm{fat}\;\mathrm{mass}\;\left[\mathrm{kg}\right]}{{\mathrm{body}\;\mathrm{height}\;[m}^{2}]}}^{}$$

In schemes of calculated models, explanations of associations between different variables, and overall interpretations, the term FAT was used to generalize the description of all fatness variables (BFM, FMI, and BMI).

### 2.4. Cardiovascular Fitness

The physical fitness index (FI) that defines cardiovascular fitness (CRF) was determined using the Harvard Step Test. Before each test, a Polar H1 heart rate monitor was fitted to each student. Participants stepped up and down on a 41.3 cm high stool at a pace of 30 cycles (1 cycle: right leg starting step up, left added, right leg step down, left added) per minute, with a metronome set at 120 bpm. The exercise continued for up to 300 s, less if participants became fatigued. Resting heart rate, changes during exercise, and recovery pulse was measured. Recovery pulse was recorded within 1.5 min of recovery. Heart rate monitors sampled participants pulses at 5-s intervals and were transmitted to a smartwatch (Polar, Polar Electro; Kempele, Finland). FI was calculated using the following formula [30]:

FI = (100 × L)/(5.5 × p),(3)

where: L = duration of the test in seconds, L < 300 s, and *p* = heart rate within 1.5 min after the subject stopped the test.

Due to performing the study in school settings (physical education lessons), the intensity of the exercises was adjusted to the study subjects who do not participate in any sports type. The established HIIT intensity was safe in order to avoid any side effects such as dizziness or faintness. Moreover, the effort intensity was acceptable for the pupils; therefore, they did not refuse participation. Simultaneously, it was possible to achieve broad positive effects associated with the HIIT intervention [20].

### 2.5. Blood Pressure Measurements

In this work, we used the results of resting blood pressure after a 10-week intervention. Automatic Blood Pressure Monitor—Omron BP710 measured blood pressure parameters. Participants were asked to sit quietly for 10 min before measurements. Readings of resting SBP and DBP were taken three times in 10-min intervals. The mean value of the three measurements was recorded into the database. 

### 2.6. Identifying Individuals with Hypertension—Exclusion Criteria

As aforementioned, the assumption in this work was non-hypertensive blood pressure. The procedure of classifying the adolescents based on the systolic and diastolic blood pressure measurements met the criteria contained in the National High Blood Pressure Education Program (NHBPEP) Working Group published in the 4th Report (IVR) [31]. According to IVR, in hypertension state, average SBP and DBP are over the 95th percentile 

Proper and adequate percentiles used in this work were calculated for Polish adolescents [32]. We used the following (mm of Hg) values in boys: C95/SB*P* = 133, C95/DB*P* = 82. Similarly, we adopted girls’ values: C95/SB*P* = 130, C95/DB*P* = 81. 

### 2.7. Intervention

The above description of the HIIT intervention was presented elsewhere [20]. Here, briefly, a HIIT program was conducted in one (out of three) lesson per week for ten weeks. Ten-minute warm-ups consisting of jogging and stretching exercises preceded the main exercises. The central part of the program was the HIIT intervention with a total time of 14 min. The HIIT intervention consisted of three cycles based on the Tabata protocol (8 rounds of 20 s work/10 s rest). Between processes was a 1-min break. All the exercises were played on a screen to ensure that workout and rest were implemented accurately.

Evaluation of the intensity of the workout was based on the Tanaka formula:

HRmax = 208 − 0.7 × “age” (age = 16 years in this study)(4)

Using the Polar H1 (Polar Electro, Kempele, Finland), participants’ heart rate was measured and was established in the range of 75–80% HRmax (145–157 heartbeats/min) to perform the HIIT. 

### 2.8. Statistical Analysis

Descriptive statistics of the measurements: FI, BFM, FMI, BMI and SBP, DBP are presented as means, SDs, and 95% CI and were calculated separately for boys and girls (Table 1). Mediation analyses were performed using the procedure described by Baron and Kenny [27], accompanying Jamovi’s Advanced Mediation Models 1.0.4 module (Jamovie, v. 1.6, 2020, The Jamovi Project, Sydney, Australia). Mediation models were calculated. A non-parametric sampling procedure, bootstrapping, has been advocated to obtain percentile-based confidence limits [33]. This procedure provides total and specific indirect effects (through the proposed mediator: fatness) of the predictor (CRF) on outcomes (SBP and DBP). The indirect impact was calculated using 10,000 bootstrap samples for the bootstrap confidence intervals (CI) corrected for bias. An indirect effect is considered statistically significant if the CI established (95% CI) does not include 0. If the CI contains the value 0, the null hypothesis demonstrates that the indirect effect equals 0, i.e., there is no association between the variables considered [27]. The significance level was set at *p* = 0.05. Statistica V. A 13.0 statistical package (Tibco, 2020,Statsoft Poland, Cracow, Poland) was used to calculate descriptive statistics.

## 3. Results

Table 1 displayed baseline descriptive statistics CRF (FI points), BFM, FMI, and BMI, as well as postintervention SBP and DBP. 

Both sexes—male and female—were in the normal range of body mass index and the level of body fat, suggesting healthy fatness (not overweight nor obesity). Dispersion of all fatness variables suggested very consistent groups. Whereas both groups, boys and girls, received very low results of fitness index locating their cardiorespiratory fitness in the very poor category. The mean value of systolic blood pressure was lower in boys, while girls received a mean value very close to standard 120 bps, while diastolic blood pressure was lower in girls than boys (Table 1).

### 3.1. CRF and BP Associations Analysis

One of the study’s objectives was to investigate the effects of the baseline CRF on post-intervention SBP and DBP in adolescents. Calculated total effects, extracted from moderated mediation model, was an indicator of the influence of the CRF on SBP and DBP.. Results calculated for males and females separately confirmed the significant and negative impact of CRF on SBP (boys: B = −0.603, *p* = 0.010; girls B = −0.394, *p* = 0.037), but not significant (though negative) on DBP (boys: B = −0.055, *p* = 0.829; girls B = −0.184, *p* = 0.373). These results suggested that lower CRF at the baseline is significantly associated with higher post-intervention systolic blood pressure and not significantly with diastolic blood pressure. 

Although Baron and Kenny originally suggested the statistical significance of the relationship between the independent variable (IV) and dependent variable (DV) to start mediation analysis, this step is controversial. Shrout and Bolger [34] proved the possibility of moving forward to the following analysis step, even if the association between IV and DV is insignificant. Thus, the mediation analysis was continued for both cardiovascular parameters, despite the insignificant results revealed for DBP (aforementioned insignificant total effect). 

### 3.2. Sex Moderation Analysis

**Figure 2:**
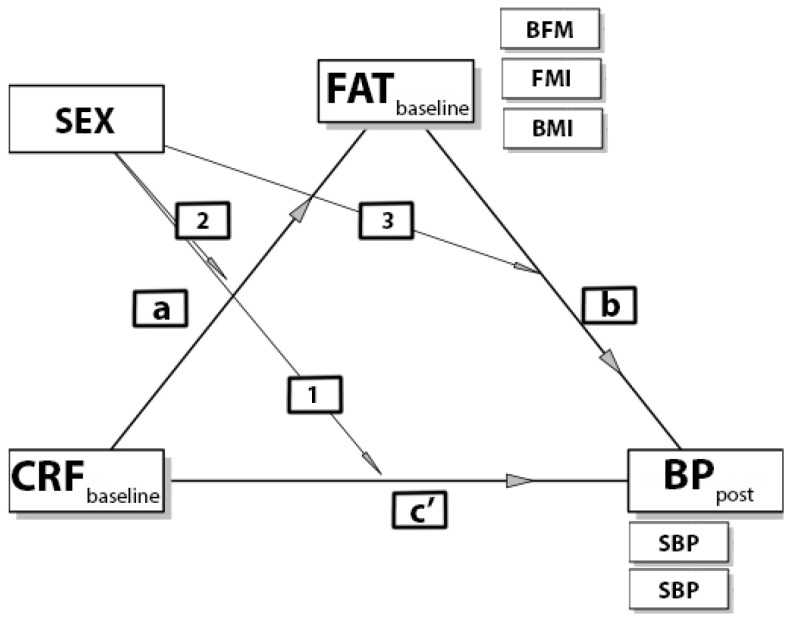
Hypothesized moderation model of potential effects of sex (moderator) on the relationship between CRF and BP through FAT (mediator). Numbers indicate effects of moderation on mediation model’s elements: 1—sex on indirect influence of CRF on BP, 2—sex on the component: CRF effect on FAT (BFM, FMI, BMI), 3—sex on the component: FAT (BFM, FMI, BMI) effect on BP. Letters indicate effects of mediation: c’—direct effect, a—path an of indirect impact, b—path b of indirect impact.

Another study’s objective was to assess the potential moderation role of sex in mediation models. This effect was examined before the main mediation analysis, which allowe the adoption of a proper analytic strategy (total lack of interaction—mediation analysis in the whole group, interaction in any path—mediation analysis in boys and girls, separately). The hypothesized model of moderated mediation is presented in Figure 2. 

Moderation effects were represented by statistically significant interactions between categories of the moderator (sex: boys and girls) and paths of mediation model: 1. direct effect of CRF on BP; 2. indirect effect’s component: CRF on FAT; 3. indirect effect’s component: FAT on BP. Suppose there was a significant interaction (*p* > 0.05); this meant differences in the element of the mediation model between both sexes. Lack of significant interaction (*p* > 0.05) meant the same effect in both sexes’ elements of the mediation model. 

Calculated *p*-values for interactions are presented in Table 2. The results of the moderation analysis identified interactions practically in each model (except in a model with BFM as a mediator in the model regarding DBP). It meant significant differentiation of the mediation models between both sexes. Thus, the moderation role of the sex was confirmed. Therefore, the next stage was mediation analysis in both groups: boys and girls, separately.

### 3.3. Mediation Analysis

The main objective of this work was to examine whether baseline CRF affected resting BP after intervention through the mediation of fatness. The mediation effect occurs when the independent variable’s direct effect (path c’) on the dependent variable disappears after including the mediator. Both paths (a and b) related to the mediator are significant. Analysis was conducted separately for each potential mediator (BFM, FMI, BMI). Results are presented in Table 3, Table 4 and Table 5.

As already mentioned above, the total effect (path c) of baseline CRF on post-intervention SBP was statistically significant, and, conversely, the same effect on DBP was not statistically significant. Therefore, the mediation effect that occurs was determined by the statistical significance of the indirect effect (paths ab) and the insignificance of the direct effect (path c’).

Mediation results with BFM as a mediator are shown in Table 3. In boys, CRF at baseline was negatively associated with baseline BFM (path a, *p* = 0.009), and BFM was positively related to post-intervention SBP (path b, *p* = 0.009). Moreover, after including the mediator, the association between CRF and SBP (direct effect—path c’) was no longer significant (*p* = 0.076). Therefore, all hypotheses were confirmed—mediation effect was present, and preintervention level of BFM acted as mediator. Conversely, there was no mediation effect of the BFM in the model constructed for postintervention DBP. Baseline CRF and BFM were not associated, as well as there was no significant connection between BFM and DBP. Considering non-significant total effect and direct effect, no relationship between CRF and DBP can be concluded

In girls, the mediation effect of BFM in the relationship between CRF and SBP was not observed. The statistically significant total effect was still significant after introducing BFM as a mediator. Moreover, both indirect components (paths a and b) were insignificant. Thus, BFM was no mediator in girls, but baseline CRF independently affected post-intervention SBP. In the case of DBP, similarly to boys, the mediation effect of BFM on DBP was not observed. 

The results of mediation analysis confirmed the aforementioned moderating role of sex in the case of SBP (boys—presence of mediation effect, girls—mediation effect not present) and lack moderating effect in case of DBP (both boys and girls, no mediation effect).

Table 4 shows the mediation results, including FMI as a mediator. The picture of associations between the tirade of variables with FMI as a mediator very well imitated associations observed in the model with BFM. 

In boys, the initial significant total effect of baseline CRF (negatively associated with postintervention SBP) was no longer significant after including FMI as mediator (direct effect—path c’, *p* = 0.057). Moreover, CRF was significantly and negatively related to FMI (path a, *p* = 0.029) and FMI was significantly and positively associated with SBP (path b, *p* = 0.007). Thus, also, in this case, all hypotheses were confirmed. Mediation effect was indicated, and FMI (similarly to BFM) played the role of a mediator. In the case of postintervention DBP, both total and direct effects of mediation were statistically significant. Both paths of the indirect effect of mediation (a and b) were not significant. Therefore, FMI was no mediator. Moreover, there were no associations between CRF, FMI, and post-intervention DBP.

After including FMI in mediation models concerning SBP and DBP, there was no mediating effect in girls. 

Additionally, in the case of FMI moderation, the role of sex was confirmed concerning SBP. The mediation effect was present in boys, while it was not present in girls. In contrast, there was no moderation effect of sex concerning DBP (neither mediation effect in boys nor girls).

Table 5 presents the results of the mediation analysis, including BMI. In both sexes, male and female, the initial significant impact of the baseline CRF on postintervention resting SBP (total effect) was still significant after including BMI as a mediator (direct effect—path c’, *p* = 0.019 in boys, *p* = 0.036 in girls). In contrast, both indirect paths (a and b) were insignificant. Therefore, there was no mediation effect. In the case of DBP, there were no mediation effects, both in boys and girls.

## 4. Discussion

This study showed a statistically significant inverse relationship between baseline cardiorespiratory fitness and post-intervention systolic blood pressure. The associations between cardiorespiratory fitness and postintervention diastolic blood pressure were not observed (either in boys or girls). After including the third variable, examining the character of the relations between CRF and postintervention SBP revealed the mediation effect of the BFM and FMI in boys but not in girls. Thus, a moderated mediation was observed, which was manifested in the fact that the influence of the baseline CRF on post-intervention SBP in girls was autonomous and independent of fatness. There was no mediation in the case of BMI, either in boys or girls. These findings expand current knowledge about the importance of fatness as a feature mediating negative associations between CRF and BP after the intervention program.

The present study, similar to previous ones [35,36], showed negative associations between cardiorespiratory fitness and systolic blood pressure. It suggested a relation: the higher the CRF level, the lower blood pressure level, and vice versa. Cardiorespiratory fitness is strongly related to physical activity. Regular physical exercise carried out over a long period helps to improve CRF [26]. It was documented that the longitudinal association between CRF and BP was based on the physiological adaptations of regular physical activity, and CRF has been shown to improve through sufficient PA [37]. However, associations between baseline CRF and post-intervention BP were assessed in this study. The potential ways of influence and the mechanisms acting in such a relationship may be a lot. Indeed, the effects of CF on BP are based on vascular and endocrine functions [38]. The beneficial effects of CRF on BP were explained by mechanisms based on decreased vascular peripheral resistance, together with reduced sympathetic nervous system activity, neurohormonal changes, and vascular remodelling in vessels, muscle, and adipocytes [39,40]. 

CRF and fatness are considered health predictors and cardiovascular disease risk factors [41]. Very strong associations between both of them were also observed. Previous studies documented a relationship between CRF and fatness and cardiovascular risk factors in adolescents [42,43]. However, to date, which is more critical for health has not been decided [41]. In many works, fatness is assessed indirectly through body mass index. There is evidence that BMI is a cardiometabolic risk factor significantly associated with health [44]. Therefore, the results showed negative associations between CRF and BMI. Higher BMI was related to lower CRF.

On the contrary, the results of this work showed no relationship between BMI and CRF (it was defined in the path of mediation analysis). It is difficult to interpret this result. Probably, it depended on a small number of participants. Considering that there was the effect of CRF and BFM and FMI, it can be concluded that BMI is a less sensitive body fat measurement for mediating procedure than total body fat mass or fat mass index, particularly concerning a small group of individuals. 

Fatness, particularly a very high level of fatness, is strongly linked to blood pressure. The dependence is positive—the higher the fat level, the higher the blood pressure level. Thus, overweight and obesity are very closely linked to hypertension. Obesity causes functional and structural changes in the microcirculation that impair microvascular functions underlying elevated blood pressure [45]. The explanation was that obesity-related microvascular dysfunction might increase peripheral vascular resistance and consequently contribute to the development of hypertension [45,46]. The results of this work are consistent with previous observations. The strong, significant, and positive relationships between body fat mass and fat mass index were observed for cardiovascular functions—systoles and diastoles. 

The main finding in this study is the mediation effect of fatness (BFM and FMI, but not BMI) in the relationship between CRF and SBP. It is difficult to explain why the mediation effect was not observed in DBP. Moreover, the mediation effect was observed only in males. Thus, it can be concluded that moderated mediation was revealed. This meant that categories of the factor moderating phenomena changed the final results. In this case, sex is a moderator; therefore, the relationship in males is different than in females. 

The mediation mechanism assumes that the independent variable influences the mediator, and the mediator affects the dependent variable. So, the independent variable’s total effect is divided into indirect effects through a mediator. Based on the mechanism mentioned above, our results suggested that CRF at baseline probably ameliorates the fatness level and fatness and affects blood pressure. Our results are convergent to previous studies with mediation analysis. Inverse associations between CRF and BMI, body fat percentage (BFP) or waist-to-hip ratio (WHR), as well as an inverse relationship between these fatness measurements and blood pressure, with simultaneous lack of influence of the CRF on BP (total effect), were observed [26,38]. The mediation role of fatness and the mechanisms underlying the associations have attempted to be explained in several studies. The suggestion was, among others, that CRF impacted fatness through energy balance and increased capacity of fat oxidation, since CRF is associated with mitochondrial volume [47]. On the other hand, the association between fatness and BP may be related to the occurrence of chronic low-grade inflammation, increased oxidative stress, or altered adipokine secretion (related particularly to adolescents), which can lead to arterial stiffness and endothelial dysfunction underlying elevated blood pressure [48,49,50,51]. Those previous results support our findings, suggesting that higher CRF in adolescents leads to improved fatness levels and, in consequence, affects BP. 

Our mediation analysis gave a new insight into understanding the relationship between fatness, CRF, and SBP. It can be suggested that BFM and FMI are more sensitive measurements than BMI to detect the mediation role of fatness in associations between independent and dependent variables (CRF and SBP in this case). However, the mediation role of fatness was moderated by sex; mediation was observed only in males. The lack of mediation, but the direct association between CRF and SBP, independently of fatness, suggested the necessity of enhancing the importance of aerobic fitness as a health indicator in girls.

The strength of this study includes its intervention design. Such a project gave new insight into how high-intensity interval training may affect blood pressure and how this effect depends on the level of fatness—the interaction design allowed to make cause–effect interferences. 

This study has several limitations. The first is its small number of participants. In addition, participants were homogeneous concerning age. Groups of different ages (particularly biological age with maturity offset under control) should be considered. Another limitation is the lack of other relevant covariates such as lifestyle elements (e.g., nutrition, smoking habits, physical activity). Findings cannot be extended to the entire population of polish youth as the sample was not nationally representative. Measurement of CRF should include the indirect evaluation of VO2max with an accurate field test.

## 5. Conclusions

In conclusion, the results of this intervention study showed that baseline cardiorespiratory fitness impacted only post-intervention systolic blood pressure, but there was no significant influence on diastolic blood pressure. Moderation analysis, carried out initially, revealed sex as a factor modifying mediation models. In girls, CRF acted autonomously, without the mediation of fatness, whereas in boys, the influence of the CRF on the SBP was not autonomous, but fatness acted as a complete mediator in the inverse associations between CRF and SBP, after high-intensity interval training in adolescents. Thus, the positive effect of the intervention program (in this case, HIIT) on systolic blood pressure was mainly boys with better baseline cardiorespiratory fitness and relatively low baseline fatness (low body fat mass and proportionally low fat to height). 

Therefore, the suggestion that optimal cardiorespiratory fitness and fatness levels were essential to maintain balanced (far from hypertension) post-intervention systolic blood pressure contributes to the comprehension of the relationship between the relationship and the triad of main factors of cardiovascular health. Findings from this work can be considered in preparing interventions focusing on the effects of cardiovascular health in youth. Interventions should be tailored separately for males and females, and effective methods for both cardiovascular functions should be sought. 

Body fat mass and fat mass index proved to be more accurate measurements and more perfect indicators for the mediating role of fatness than BMI.

## Figures and Tables

**Table 1:** Descriptive statistics of the baseline and postintervention cardiorespiratory fitness (CRF), body fat mass (BFM), fat mass index (FMI), body mass index (BMI), and blood pressure (systole—SBP and diastole—DBP).

	Boys		Girls	
Variable	Mean (±SD) 95%CI		Mean (±SD) 95%CI	
	pre	Post	pre	Post
Cardiorespiratory fitness [FI pts]	43.94 (4.67) 42.13–45.75	47.48 (4.64) 45.68–49.28	44.48 (5.05) 42.77–46.19	45.09 (3.58) 43.88–46.31
body fat mass (BFM) [kg]	11.74 (8.42) 8.47–15.00	9.02 (5.57) 6.86–11.18	14.98 (4.07) 13.60–16.35	14.98 (4.42) 13.48–16.48
fat mass index (FMI) [kg/m^2^]	3.75 (2.63) 2.73–4.77	2.87 (1.63) 2.23–3.50	5.45 (1.49) 4.94–5.95	5.45(1.48) 4.95–5.96
body mass index (BMI) [kg/m^2^]	20.71 (3.55) 19.33–22.09	20.63 (3.26) 19.36–21.89	20.51 (1.97) 19.85–21.18	20.55 (2.07) 19.85–21.26
systolic blood pressure (SBP) [mmHg]	123.78 (13.73) 118.46–129.11	114.21 (7.11) 111.46–116.97	116.83 (7.46) 114.30–119.35	121.33 (5.35) 119.52–123.14
diastolic blood pressure (DBP) [mmHg]	70.96–76.75 73.85 (7.46)	72.04 (5.61) 69.86–74.21	71.66 (7.04) 69.28–74.05	67.53 (6.73) 65.25–69.81

**Table 2:** Interaction terms of sex on the three arms of mediation models (1–3 on Figure 1) in the relationship between CRF and BP (SBP and DBP) through FAT (BFM, FMI, BMI)—*p*-values.

		Direct Effect	Components	
Mediator	BP Parameter	(1) CRF⇨BP	(2) CRF⇨FAT	(3) FAT⇨BP
BFM	SBP	0.972	0.062	**0.045**
	DBP	0.638	0.062	0.913
FMI	SBP	0.880	**0.044**	0.341
	DBP	**0.051**	0.094	0.624
BMI	SBP	**< 0.001**	0.153	0.602
	DBP	**< 0.001**	0.153	0.858

Note: CRF—cardiorespiratory fitness, FAT—fatness, BFM—body fat mass, FMI—fat mass index, BMI—body mass index, BP—blood pressure, SBP—systolic blood pressure, DBP—diastolic blood pressure. Statistically significant values in bold.

**Table 3:** The mediation effects of the baseline BFM: total (path c), direct (path c’), and indirect (ab) with its components (paths a and b).

BP Parameter	Effect	Boys				Girls			
		B	95% CI		*p*	B	95%	CI	*p*
SBP	Total (c)	−0.60	−1.06	−0.15	**0.010**	−0.39	−0.77	−0.02	**0.037**
	Direct (c’)	−0.41	−0.87	0.04	0.076	−0.4	−0.76	−0.05	**0.025**
	Indirect (ab)	−0.19	−0.39	0.01	0.065	0.01	−0.05	0.07	0.802
component CRF⇒BFM (a)		−0.64	−1.12	−0.16	**0.009**	−0.05	−0.44	0.34	0.799
	BFM⇒SBP (b)	0.30	0.07	0.52	**0.009**	−0.16	−0.38	0.06	0.163
DBP	Total (c)	−0.06	−0.55	0.44	0.829	−0.18	−0.59	0.22	0.373
	Direct (c’)	−0.02	−0.54	0.50	0.947	−0.18	−0.58	0.22	0.380
	Indirect (ab)	−0.04	−0.20	0.13	0.653	0.00	−0.04	0.03	0.812
component CRF⇒BFM (a)		−0.64	−1.12	−0.16	**0.009**	−0.05	−0.44	0.34	0.799
	BFM⇒DBP (b)	0.06	−0.19	0.31	0.648	0.09	−0.17	0.34	0.500

Note: B—estimate regression, CI—confidence coefficient index, CRF—cardiorespiratory fitness, BFM—body fat mass, BP—blood pressure, SBP—systolic blood pressure, DBP—diastolic blood pressure. Statistically significant values in bold.

**Table 4:** The mediation effects of the baseline FMI: total (path c), direct (path c’), and indirect (ab) with its components (paths a and b).

BP Parameter	Effect	Boys				Girls			
		B	95% CI		*p*	B	95%	CI	*p*
SBP	Total (c)	−0.60	−1.06	−0.15	**0.010**	−0.39	−0.77	−0.02	**0.037**
	Direct (c’)	−0.44	−0.89	0.01	0.057	−0.39	−0.75	−0.04	**0.029**
	Indirect (ab)	−0.16	−0.35	0.03	0.090	0.00	−0.04	0.03	0.980
component CRF⇒FMI (a)		−0.18	−0.33	−0.02	**0.029**	0.00	−0.13	0.13	0.980
	FMI⇒SBP (b)	0.93	0.25	0.32	**0.007**	0.27	−0.40	0.94	0.431
DBP	Total (c)	−0.06	−0.55	0.44	0.829	−0.18	−0.59	0.22	0.373
	Direct (c’)	−0.02	−0.52	0.47	0.922	−0.19	−0.58	0.20	0.349
	Indirect (ab)	−0.03	−0.16	0.10	0.659	0.00	−0.17	0.17	0.980
component CRF⇒FMI (a)		−0.18	−0.33	−0.02	**0.029**	0.00	−0.13	0.13	0.980
	FMI⇒DBP (b)	0.17	−0.57	0.91	0.653	−1.31	−2.05	−0.56	**<0.001**

Note: B—estimate regression, CI—confidence coefficient index, CRF—cardiorespiratory fitness, FMI– fat mass index, BP—blood pressure, SBP—systolic blood pressure, DBP—diastolic blood pressure. Statistically significant values in bold.

**Table 5:** The mediation effects of the baseline BMI: total (path c), direct (path c’), and indirect (ab) with its components (paths a and b).

BP Parameter	Effect	Boys				Girls			
		B	95% CI		*p*	B	95%	CI	*p*
SBP	Total (c)	−0.60	−1.06	−0.15	**0.010**	−0.39	−0.77	−0.02	**0.037**
	Direct (c’)	−0.55	−1.01	−0.09	**0.019**	−0.39	−0.76	−0.03	**0.036**
	Indirect (ab)	−0.05	−0.17	0.07	0.379	0.00	−1.07	1.06	0.994
component CRF⇒BMI (a)		−0.17	−0.35	0.01	0.065	0.00	−0.15	0.15	0.994
	BMI⇒BP (b)	0.31	−0.30	0.92	0.317	7.23	6.62	7.84	**<0.001**
DBP	Total (c)	−0.06	−0.55	0.44	0.829	−0.18	−0.59	0.22	0.373
	Direct (c’)	−0.11	−0.60	0.39	0.672	−0.17	−0.56	0.23	0.408
	Indirect (ab)	0.05	−0.07	0.18	0.412	−0.02	−4.74	4.70	0.994
component CRF⇒BMI (a)		−0.17	−0.35	0.01	0.065	0.00	−0.15	0.15	0.994
	BMI⇒BP (b)	−0.31	−0.96	0.35	0.360	31.97	31.32	32.63	**<0.001**

Note: B—estimate regression, CI—confidence coefficient index, CRF—cardiorespiratory fitness, BMI—body mass index, BP—blood pressure, SBP—systolic blood pressure, DBP—diastolic blood pressure. Statistically significant values in bold.

## Data Availability

The data presented in this study are available on request from the corresponding author.
